# Supplementary material for: The Forward and Lateral Tilt Angle of the Neck and Trunk Measured by Three-Dimensional Gait and Motion Analysis as a Candidate for a Severity Index in Patients with Parkinson’s Disease
Source: Neurol Int. 2022 Sep 13;14(3):727–37. doi: 10.3390/neurolint14030061 (PMC9504699; doi:10.3390/neurolint14030061)
Supplement: Supplementary file 1 [file neurolint-14-00061-s001.zip › neurolint-1859657-supplementary.pdf]

Table S1. MDS-UPDRS Score Sheet

Part Ⅰ		Part Ⅲ	
1.1	Cognitive impairment	3.1	Speech
1.2	Hallucinations and psychosis	3.2	Facial expression
1.3	Depressed mood	3.3	Rigidity
1.4	Anxious mood		-Neck, RUE, LUE, RLE, LLE
1.5	Apathy	3.4	Finger tapping-Right, Left hand
1.6	Features of DDS	3.5	Hand movements-Right, Left hand
1.7	Sleep problems	3.6	Pronation-supination movements
1.8	Daytime sleepiness		- Right, Left hand
1.9	Pain and other sensations	3.7	Toe tapping-Right, Left foot
1.10	Urinary problems	3.8	Leg agility-Right, Left leg
1.11	Constipation problems	3.9	Arising from chair
1.12	Light headedness on standing	3.10	Gait
1.13	Fatigue	3.11	Freezing of gait
Part Ⅱ		3.12	Postural stability
2.1	Speech	3.13	Posture
2.2	Saliva and drooling	3.14	Global spontaneity of movements
2.3	Chewing and swallowing	3.15	Postural tremor-Right, Left hand
2.4	Eating tasks	3.16	Kinetic tremor-Right, Left hand
2.5	Dressing	3.17	Rest tremor amplitude
2.6	Hygiene		-RUE, LUE, RLE, LLE, Lip/jaw
2.7	Handwriting	3.18	Constancy of rest
2.8	Doing hobbies and other activities	Part Ⅳ	
2.9	Turning in bed	4.1	Time spent with dyskinesias
2.10	Tremor	4.2	Functional impact of dyskinesias
2.11	Getting out of bed	4.3	Time spent in the OFF state
2.12	Walking and balance	4.4	Functional impact of fluctuations
2.13	Freezing	4.5	Complexity of motor fluctuations
		4.6	Painful OFF-state dystonia

Legend：DDS = Dopamine Dysregulation Syndrome, RUE = right upper extremity, LUE = left upper extremity, RLE = right lower extremity, LLE = left lower extremity.
